# Genomic landscape of colorectal cancer in Japan: clinical implications of comprehensive genomic sequencing for precision medicine

**DOI:** 10.1186/s13073-016-0387-8

**Published:** 2016-12-22

**Authors:** Masayuki Nagahashi, Toshifumi Wakai, Yoshifumi Shimada, Hiroshi Ichikawa, Hitoshi Kameyama, Takashi Kobayashi, Jun Sakata, Ryoma Yagi, Nobuaki Sato, Yuko Kitagawa, Hiroyuki Uetake, Kazuhiro Yoshida, Eiji Oki, Shin-ei Kudo, Hiroshi Izutsu, Keisuke Kodama, Mitsutaka Nakada, Julie Tse, Meaghan Russell, Joerg Heyer, Winslow Powers, Ruobai Sun, Jennifer E. Ring, Kazuaki Takabe, Alexei Protopopov, Yiwei Ling, Shujiro Okuda, Stephen Lyle

**Affiliations:** 1 Division of Digestive and General Surgery, Niigata University Graduate School of Medical and Dental Sciences, 1-757 Asahimachi-dori, Chuo-ku, Niigata City, Niigata 951-8510 Japan; 2 Niigata Cancer Center Hospital, 15-3 Kawagishi-cho 2-Chome, Chuo-ku, Niigata City, Niigata 951-8566 Japan; 3 Department of Surgery, Keio University School of Medicine, 35 Shinano-machi, Shinjyuku-ku, Tokyo, 160-8582 Japan; 4 Department of Chemotherapy and Oncosurgery, Tokyo Medical and Dental University, 1-5-45, Yushima, Bunkyo-ku, Tokyo, 113-8510 Japan; 5 Department of Surgical Oncology, Gifu University School of Medicine, 1-1 Yanagido, Gifu, 501-1194 Japan; 6 Department of Surgery and Science, Graduate School of Medical Sciences, Kyushu University, 3-1-1 Maidashi, Higashi-ku, Fukuoka 812-8582 Japan; 7 Digestive Disease Center, Showa University Northern Yokohama Hospital, 35-1 Chigasaki-chuo, Tsuzuki-ku, Yokohama, 224-8503 Japan; 8 Diagnostics Research Department, Life innovation Research Institute, Denka innovation center, Denka Co., Ltd., 3-5-1 Asahi-Machi, Machida-City, Tokyo 194-8560 Japan; 9 KEW, Inc, 840 Memorial Drive, 4th floor, Cambridge, MA 02139 USA; 10 Breast Surgery, Roswell Park Cancer Institute, Elm & Carlton Streets, Buffalo, NY 14263 USA; 11 Department of Surgery, University at Buffalo, The State University of New York, Jacobs School of Medicine and Biomedical Sciences, Buffalo, NY USA; 12 Division of Bioinformatics, Niigata University Graduate School of Medical and Dental Sciences, 1-757 Asahimachi-dori, Chuo-ku, Niigata City, Niigata 951-8510 Japan; 13 University of Massachusetts Medical School, 55 Lake Avenue North, Worcester, MA 01655 USA

**Keywords:** Colorectal cancer, Precision medicine, Ethnicity, Japanese, Comprehensive genomic sequencing, Actionable driver mutation, Hypermutation

## Abstract

**Background:**

Comprehensive genomic sequencing (CGS) has the potential to revolutionize precision medicine for cancer patients across the globe. However, to date large-scale genomic sequencing of cancer patients has been limited to Western populations. In order to understand possible ethnic and geographic differences and to explore the broader application of CGS to other populations, we sequenced a panel of 415 important cancer genes to characterize clinically actionable genomic driver events in 201 Japanese patients with colorectal cancer (CRC).

**Methods:**

Using next-generation sequencing methods, we examined all exons of 415 known cancer genes in Japanese CRC patients (*n* = 201) and evaluated for concordance among independent data obtained from US patients with CRC (*n* = 108) and from The Cancer Genome Atlas-CRC whole exome sequencing (WES) database (*n* = 224). Mutation data from non-hypermutated Japanese CRC patients were extracted and clustered by gene mutation patterns. Two different sets of genes from the 415-gene panel were used for clustering: 61 genes with frequent alteration in CRC and 26 genes that are clinically actionable in CRC.

**Results:**

The 415-gene panel is able to identify all of the critical mutations in tumor samples as well as WES, including identifying hypermutated tumors. Although the overall mutation spectrum of the Japanese patients is similar to that of the Western population, we found significant differences in the frequencies of mutations in ERBB2 and BRAF. We show that the 415-gene panel identifies a number of clinically actionable mutations in KRAS, NRAS, and BRAF that are not detected by hot-spot testing. We also discovered that 26% of cases have mutations in genes involved in DNA double-strand break repair pathway. Unsupervised clustering revealed that a panel of 26 genes can be used to classify the patients into eight different categories, each of which can optimally be treated with a particular combination therapy.

**Conclusions:**

Use of a panel of 415 genes can reliably identify all of the critical mutations in CRC patients and this information of CGS can be used to determine the most optimal treatment for patients of all ethnicities.

**Electronic supplementary material:**

The online version of this article (doi:10.1186/s13073-016-0387-8) contains supplementary material, which is available to authorized users.

## Background

Cancer remains the leading cause of death worldwide with colorectal cancer (CRC) among the most common indications, accounting for 700,000 deaths per year [1]. Utilizing next-generation sequencing technology, projects such as The Cancer Genome Atlas (TCGA) and others have profiled genomic changes in several cancer types including CRC [2–9]. The ultimate goal of cancer genome profiling is to enable precision medicine, the tailoring of treatments based on unique genomic changes of each patient’s individual tumor. For instance, the importance of genomic evaluation of RAS and RAF for advanced CRC patients has been widely accepted, since it has been revealed that tumors with RAS or RAF mutations show resistance to anti-EGFR therapies [10]. Initially, mutations in these genes were found to occur in “hot-spots” (i.e. KRAS codon 12, 13, or BRAF V600E) [11–13], however, whole exome sequencing (WES) has revealed that mutations outside of hot-spots can also influence therapeutic responses [14, 15]. Yet, WES may not be practical in the clinical setting due to its high cost, shallow sequencing depth, and excessive information about variants/genes of unknown significance [16, 17]. Although sequencing studies of CRC have been reported [4, 18–20], tumors from Asian populations have not been the subject of comprehensive evaluation. We now report the results from the analysis of 201 Japanese CRC patients.

Since all of the reported studies examined the mutational spectrum using WES, and WES is clinically expensive and time-consuming, we hypothesized that sequencing a panel of cancer-associated genes would identify essentially all actionable genomic driver mutations and further determine mutational burden in CRC, both of which can enable development of personalized treatment strategies. In the current study, we tested this hypothesis utilizing a 415-gene panel designed for solid tumors at a very high depth of coverage (~500×) in Japanese patients (*n* = 201 tumors) and evaluated for concordance among independent data obtained from US patients with colon cancer (*n* = 108 tumors) (J-CRC and US-CRC, respectively) and from the TCGA-CRC WES database (*n* = 224 tumors). Here, we report that comprehensive genomic sequencing (CGS) with a 415-gene panel can accurately determine high mutation burden (somatic mutation rate) and that there are differences in the frequency of mutations in ERBB2 and BRAF. Hierarchical clustering of clinical data revealed that a subset of 26 genes can classify all of the CRC patients into eight categories, each of which can be effectively treated with available drugs or drugs in development.

## Methods

### Patient cohorts and sample inclusion criteria

#### Japanese cohort

A total of 201 patients diagnosed with stage I–IV CRC according to AJCC 7th edition [21] who had curative surgery between 2009 and 2015 at Niigata University Medical and Dental Hospital or Niigata Cancer Center Hospital were enrolled (Additional file 1: Table S4). Patients with familial adenomatous polyposis, inflammatory bowel disease, or synchronous multiple CRCs were excluded.

#### US cohort

A total of 108 patients with histologically confirmed diagnosis of primary colorectal adenocarcinoma (stage I–IV) between 2014 and 2016 submitted for CGS as part of routine medical examination were included in this study. All tumor samples that had > 50% tumor content after macrodissection, as determined through routine hematoxylin and eosin (H&E) stain by an independent pathologist, were included. A full waiver of authorization under the Health Insurance Portability and Accountability Act (HIPAA) was granted to enable retrospective analyses for samples obtained without prior consent. All data were de-identified prior to inclusion in this study.

### Sequencing library preparation

For Japanese and US patient samples, archival tissue in the form of formalin-fixed, paraffin embedded (FFPE) tumor or unstained tissue sections obtained during routine biopsy and/or resection were used for analysis. An independent pathologist evaluated tumor content on H&E stained slides for each study sample to ensure > 50% tumor content was present. Where applicable, unstained slides were macro-dissected to enrich for tumor content and genomic DNA (gDNA) was extracted using BiOstic FFPE Tissue DNA Isolation Kit (Mo Bio Laboratories, Inc.). All sample prep, CGS, and analytics were performed in a CLIA/CAP-accredited laboratory (KEW Inc; Cambridge, MA, USA).

### Comprehensive genomic sequencing

FFPE gDNA (50–150 ng) was converted into libraries and enriched for the 415 genes with CANCERPLEX (KEW Inc.; Cambridge, MA, USA). CANCERPLEX is a clinically validated 415-gene panel enriched for coding regions and selected introns of genes with known association in cancer. Sequencing was performed on the Illumina MiSeq and NextSeq platforms with average 500× sequencing depth. Genomic data were then processed through a proprietary bioinformatics platform and knowledge base to identify multiple classes of genomic abnormalities including single nucleotide substitutions (SNPs), small insertions/deletions (indels), copy number variations (CNV), and translocations in *ALK*, *RET*, and *ROS1*. A threshold of 10% allelic fraction was used for SNPs and indels and thresholds of >2.5-fold (gains) and 0.5-fold (loss) were used. To assess somatic status of mutations in a tumor-only setting, we employed a filtering strategy similar to one recently published [22] with minor differences. In short, variants were deprioritized if they were present in a combination of dbSNP, 1000 Genomes, and ExAC databases (at AF > 1%). Next, allele frequencies for each mutation were used to fit a model to determine whether the variant is likely germline heterozygous or somatic. Finally, results underwent manual molecular pathologist review validating somatic versus possible germline status of a variant. Based on published and our experience, this approach allows the correct discrimination between germline and somatic variants in more than 99% of cases. Mutated burden was determined by non-synonymous SNPs present in the tumor that have population frequency of < 1% dbSNP and 1000 Genomes databases.

### Downsampling TCGA mutation data

COAD-READ mutation data for the TCGA-CRC samples (*n* = 224 samples) were downloaded from the Broad GDAC Firehose website (https://gdac.broadinstitute.org/). Similar to the 415-gene panel bioinformatics pipeline, silent mutations that were not protein altering were removed from the dataset. To compare mutation burden of the 415-gene panel to TCGA WES data, the dataset of SNPs was downsampled to the 415 genes in the panel and the mutation rate determined in the panel was calculated as mutations/Mb. To produce receiver operating characteristic (ROC) curves, genes were selected randomly to produce panels of 400, 300, 200, 100, and 50 genes. Mutation burden was calculated using only CGS panel genes and individual ROC curves were used to evaluate how well mutation burden predicted hypermutated samples. This process was repeated 100 times and average ROC curves were produced at each panel size. In addition, individual ROC curves were produced using all genes and only those genes in KEW’s CANCERPLEX panel.

### Mutation signature

Each single nucleotide variant (SNV) was classified in a matrix of the 96 possible substitutions based on the sequence context comprising the nucleotides 5′and 3′ to the position of the mutation. Mutational signatures were extracted using non-negative matrix factorization analysis with the SomaticSignatures R package [23] and plotted with ggplots R package (http://ggplot2.org/). This analysis identified complex signatures, different between hypermutated and non-hypermutated cases. Deconvolution of the complex profiles in order to identify components matching to COSMIC mutational signatures was done using deconstruct Sigs R package [24].

### Mismatch repair immunohistochemistry (MMR-IHC)

Immunohistochemistry (IHC) staining was performed on the 40 samples of Japanese CRC with highest mutation rates. Slides were stained for four mismatch repair (MMR) proteins, MLH1 (clone G168-15), MSH2 (clone FE11) MSH2 (clone BC/44), and PMS2 (clone A16-4), and were scored by two pathologists. For US clinical cases, clinical records were reviewed and results of MMR studies were recorded when available.

### Mutation analysis and visualization

Genomic data for Japanese (*n* = 201) and US patients (*n* = 108) obtained from CGS were mined in OncoPrinter (www.cbioportal.org). Pathway genes were selected based on previously published TCGA data [4] that are included in the 415-gene panel. For TCGA analyses, genomic profiles were selected in cBioPortal for mutations and putative copy-number alterations from GISTIC for which tumor sequence data are available (*n* = 224). For each pathway, the number of total uniquely altered cases was determined. Statistical significance was determined by Fisher’s exact two-tail test with a 95% confidence interval. For dsDNA break repair pathway analysis, the statistical significance of Japanese and US datasets was determined as compared to TCGA.

To align mutations with their protein domains, genomic data for Japanese, US, and TCGA datasets were analyzed in Mutation Mapper (www.cbioportal.org). Lollipop figures were generated for select genes implicated in colorectal adenocarcinoma. For BRAF and KRAS, data were further segregated by hypermutation status (hypermutated versus non-hypermutated).

### Gene clustering analysis

Mutation data from non-hypermutated J-CRC patients (*n* = 184 tumors) were extracted and clustered by gene mutation patterns. Two different sets of genes from the 415-gene panel were used for clustering: (1) 61 genes with frequent alteration in CRC; and (2) 26 genes that are clinically actionable in CRC. For this analysis, *KRAS* and *NRAS* were integrated into one gene as a RAS.

The number of common mutated genes related to donors i and j was presented as an element c_ij_ of an N × N matrix, where N is the number of non-hypermutated donors. In order to normalize the elements of this N dimension symmetric matrix into values ranging from 0 to 1, the original element was replaced by 1 / (c_ij_ + 1) that indicated the level of similarity between donors i and j. Because of this normalization, donors with more common mutated genes would more possibly come from a relatively close group. Consequently, a matrix with the normalized values between all donors was created. Hierarchical clustering of the matrix was performed for classifying donor groups with different mutated-gene patterns by Euclidean distance and Ward’s clustering. For the 26-gene set, donors were divided into eight groups based on the hierarchical clustered dendrogram, which clearly distinguished donors by the different mutated-gene patterns. On the other hand, for the 61-gene set, donors were divided into 17 groups. These clusterings were performed by software R (https://www.r-project.org/).

### Model selection of clustering

Clustering stability was evaluated by R package *clValid* for statistical and biological validation of clustering results (https://cran.r-project.org/web/packages/clValid/index.html). This method would produce the results of four stability measures called APN (average portion of non-overlap), AD (average distance), ADM (average distance between means), and FOM (figure of merit). For each index, a lower value means higher stability. We attempted clustering stabilities for combinations of different numbers of clusters obtained by cutting a dendrogram (2–12 for the 26-gene set and 2–24 for the 61-gene set) with different distance methods (“Euclidean,” “maximum,” “manhattan,” “canberra,” and “minkowski”) and clustering methods (“ward.D,” “ward.D2,” “single,” “complete,” “average,” “mcquitty,” “median,” and “centroid”). All combinations of these three parameters were evaluated and the parameters with the lowest values of each stability index were extracted. Of these, the common parameter sets with relatively lower values among the four stability indices were selected. The most appropriate cluster number, distance method, and clustering method were determined from the resulted parameter settings, taking into account that the number of donors presented in clusters (>5 donors) would be maximized as possible and the primary mutated genes would be clear. The final selected parameter settings were the Euclidean distance method and ward.D clustering in both sets and eight clusters for the 26-gene set and 17 clusters for the 61-gene set.

### Statistical analysis of clinical information

To estimate associations between mutated-gene patterns and clinical information such as sex, rectum/colon, and left/right, a two-tailed Fisher’s exact test was applied in each cluster. Additionally, in order to explore associations between mutated-gene patterns and tumor aggressiveness, seven clinical variables were dichotomized into less or more aggressive factors for colon cancer onsets in the following manner: lymphatic invasion (absence/presence), vascular invasion (absence/presence), histopathological grade (G1/G2 or G3), size of primary tumor (T1/T2 or T3/T4), spread to regional lymph node (N0 or N1/N2), distant metastasis (M0 or M1), and tumor stage (I/II or III/IV). In each cluster, two-tailed Fisher’s exact test was applied to all clinical categories by comparing the distribution in a cluster group to that of all the donors in the other groups. Note that in the case of statistical signature for 17 hypermutated donors, two-tailed Fisher’s exact test was conducted against 184 non-hypermutated donors as a reference set.

Patients were followed every 1–6 months at outpatient clinics. Medical records and survival data were obtained for all 104 Stage IV CRC patients. Among them, 46 patients received anti-EGFR therapies. Seven out of the 46 patients with surgical resection were excluded and 39 patients were included for the analysis of clinical outcomes. Tumor assessments at baseline included a computed tomography (CT) scan of the abdomen as well as of other relevant sites of the disease. Follow-up scans to assess response were obtained after cycles 1 and 2 and every two cycles thereafter. Responses were determined using RECIST 1.0. Six patients who showed progression disease before the first assessment for RECIST were excluded and 33 patients were included for waterfall plot analysis. The best calculated responses on the basis of measurable lesions were analyzed by waterfall plot.

The follow-up period for progression-free survival was defined as the interval between the date of diagnosis of metastatic disease and that of progression disease. Survival curves were constructed using the Kaplan–Meier method and differences in survival were evaluated using the log-rank test. Three out of 39 patients were excluded for Kaplan–Meier analysis based on the clustering, since each one of three patients was classified into each different subtype alone. All statistical evaluations were performed using the SPSS 22 software package (SPSS Japan Inc., Tokyo, Japan). All tests were two-sided and a *P* value < 0.05 was considered statistically significant.

While conducting the two-tailed Fisher’s exact test as above, the statistical powers of the tests were also estimated by R package *statmod* (https://cran.r-project.org/web/packages/statmod/index.html). Some clinical categories showing significant differences (*p* < 0.05) were at insufficient power levels (power < 0.8). It is known that power is related to sample size and, in other words, the power of tests could be promoted by adjusting the effect size of samples [25]. Therefore, for these significant but low-power contingency tables, we made a prediction of the number of donors that could meet a sufficient power level under the premise that the hypothetical cross-tabulations had the same cell percentages as that of 184 non-hypermutated donors. The prediction was performed for sample sizes in the range of 20–500 with increments of ten donors for each step and both *P* value and power of Fisher’s exact test were calculated for assumed contingency table at each step. By this means, a minimum effect non-hypermutated donor number was obtained and this sample size could become a reference in future studies. The statistical power calculation and prediction for the above-mentioned Fisher’s exact test were simulated 1000 times for each cross-tabulation.

### Gene-based statistical analysis

To estimate associations between genes and tumor aggressiveness, we performed Fisher’s exact test for each gene in seven clinical categories. Subsequently, significant genes with at least one clinical category (*p* < 0.05) were extracted. A matrix between the genes and the clinical categories were created based on log odds ratio for the extracted genes. Finally, the matrix was clustered by Euclidean distance and Ward’s method. In this clustering, positive and negative infinity values are replaced by 4 and −4 as pseudonumbers, respectively.

## Results

### Genomic alterations in cancer signaling pathways

**Fig. 1:**
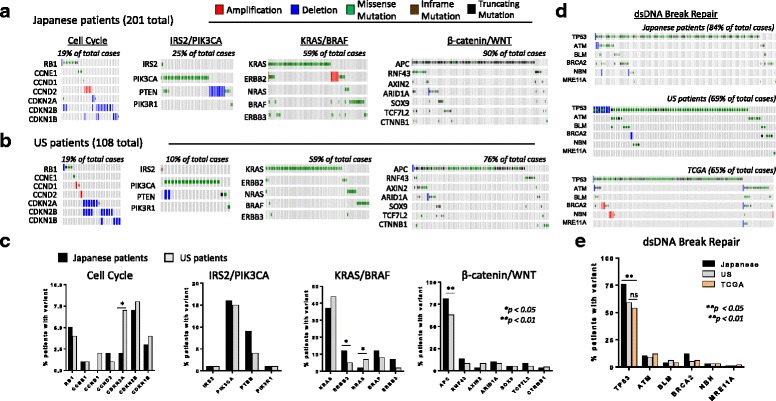
Genetic aberrations across common oncogenic pathways in CRC. Japanese patients (**a**) and US patients (**b**) were evaluated for gene alterations in the key cancer pathways. Amplification (*red*), deletion (*blue*), missense point mutations (*green*), or frameshift mutations (*brown*). Altered cases are defined as the total number of unique samples with a genetic aberration in each pathway. **c** Percent of patients with a variation for each given gene. Statistical significance was determined using Fisher’s exact test. **d** J-CRC, US-CRC, and TCGA sample data were evaluated for gene alterations in the dsDNA break repair pathway in the 415-gene panel. **e** Percent of patients with a variation for each given gene. Statistical significance was determined using Fisher’s exact test

Utilizing the CGS platform (Additional file 1: Table S1), we assessed the genes and pathways most frequently altered in the test samples (Fig. 1). We found that the same sets of alterations were generally detected by both WES and CGS. Genomic alterations in oncogenic pathways involving cell cycle, RAS/RAF, PI3K, and WNT were comparable (Fig. 1) [4]. However, we found statistically significant differences in ERBB2 (*p* < 0.05), APC (*p* < 0.001), TP53 (*p* < 0.001), CDKN2A (*p* < 0.05), and NRAS (*p* < 0.05) mutations in Japanese patients as compared with US patients (Fig. 1a–c), which may reflect epidemiological differences between the two populations [26, 27].

Given the recent recognition that tumors with DNA double-strand break repair defects (most notably *BRCA1/2* mutations) are more sensitive to PARP inhibitors [28] and the recent approval of olaparib for advanced ovarian cancer, we undertook a comprehensive analysis of the DNA double-strand break repair pathway. Currently *BRCA1/2* mutation status alone is used to identify patients for olaparib treatment; however, mutations in other genes can lead to DNA double-strand break repair defects [28, 29]. Therefore, those genes may also be useful in determining olaparib sensitivity. Excluding TP53, which is not used for selection of PARP inhibitors, we analyzed the five DNA repair pathway genes that are most commonly mutated in Japanese and US patients and compared with TCGA samples (Fig. 1d and e). We found genomic alterations in all five DNA repair genes, including *BRCA2*, which represent a significant proportion of CRC patients (26% of Japanese, 21% of US, and 19% of TCGA samples).

### Mutation rates detected by targeted sequencing with cancer gene panel

**Fig. 2:**
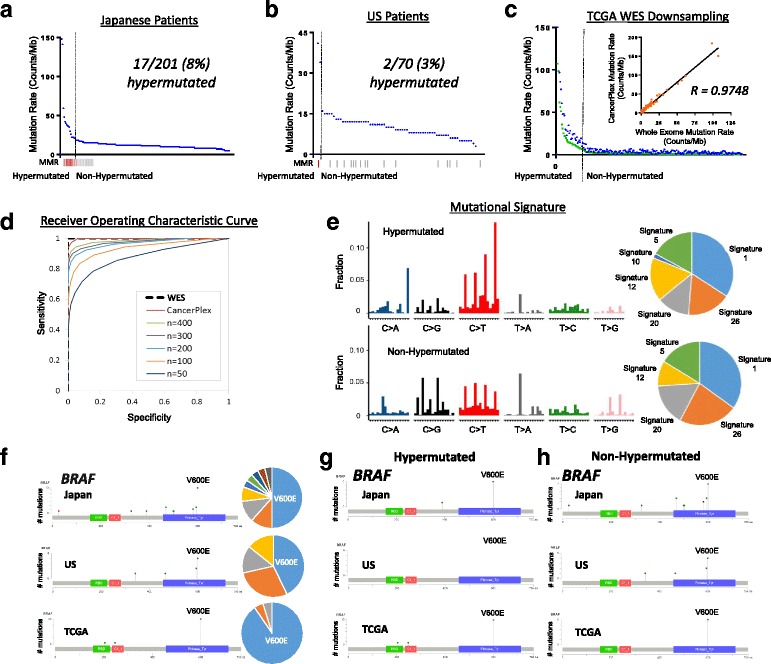
Mutation rates in Japanese and US CRC patients. Mutation rates from Japanese patients (**a**) and US patients (**b**) were determined by the number of non-synonymous SNVs in the 415-gene panel. Hypermutated and non-hypermutated cancers separated by the *dashed line. Red*, MMR-deficient; *gray*, MMR-intact; *white*, no data. **c** Data from TCGA CRC cases (*green*) were downsampled to the content of the 415-gene CGS platform (*blue*; non-synonymous SNPs). Correlation between mutation rates determined by CGS and WES (insert). **d** ROC analysis using the 415-gene CGS platform, WES, and random sets of 400, 300, 200, 100, and 50 genes as predictors of hypermutated samples (TCGA dataset). **e** Aggregated mutational signature profiles for hypermutated (*top*) and non-hypermutated cases (*bottom*). The *pie charts* represent inferred contribution of COSMIC signatures to corresponding profiles. **f** Mutations in BRAF for Japanese patients (*n* = 201), US patients (*n* = 108), and TCGA samples (*n* = 224) were aligned to protein domains. The number of mutations at each given amino acid were plotted in corresponding *pie graphs*. As shown, BRAF V600E was the highest frequency mutations in each protein. Patient samples were further plotted by mutation status: (**g**) BRAF-hypermutated, (**h**) BRAF-non-hypermutated

The clinical significance of identifying hypermutated tumors has recently been demonstrated in several studies correlating mutation burden with the development of neo-antigens and clinical response to immunotherapy drugs [4, 30–33]. We found hypermutated tumors as identified by CGS: 17 (8%) in J-CRC and two (3%) in US-CRC (Fig. 2a and b), generally correlated with DNA mismatch repair deficiency (MMR-D) as detected by standard clinical IHC evaluation for MMR proteins (MLH1, MSH2, MSH6, and PMS2). For Lynch syndrome genes, both somatic and potentially germline pathogenic mutations were included in the analysis (see “Methods”). One patient showed loss of MSH2 expression by IHC supported by genetic loss for MSH2 gene without a hypermutated phenotype while conversely two patients with the highest mutation burdens were MMR-intact and microsatellite stable but had POLE mutations, demonstrating that although often useful in predicting hypermutation status, neither MMR-D nor MSI-H alone can fully predict all hypermutated tumors. Similar analysis of US-CRC clinical cases confirmed the ability of CGS to detect hypermutated tumors, although the clinical bias to perform CGS on advanced cases of MSS-CRC in US community oncology practice may explain the low percentage of hypermutated tumors found in these samples. To further validate utility of CGS in identifying hypermutated tumors, we downsampled the TCGA WES data (*n* = 224 tumors) [4] to the subset of 415 genes in the CGS platform. This analysis not only accurately identified the hypermutated tumors (both MMR-deficient and MMR-intact) but also showed strong correlation in mutation rates between the 415-gene panel and WES (Fig. 2c). The average mutation rate detected by CGS was higher than that detected by WES reflecting the fact that the panel content was in part selected to include genes more frequently mutated in cancer. We further downsampled the TCGA data to random gene panels of descending size (400, 300, 200, 100, and 50) and determined that panels smaller than 300 genes lacked sufficient statistical power to accurately identify hypermutated cases (Fig. 2d), thus demonstrating that the CGS platform (roughly 1/2000th of the genome) is comparable to WES in generating mutation rates and to distinguish hypermutated and non-hypermutated tumors.

We further explored the utility of CGS to provide clinically meaningful patterns of mutational signatures [34] from the J-CRC cohort (Fig. 2e). Based upon the signatures described in COSMIC (http://cancer.sanger.ac.uk/cosmic)), we found that Signatures 20 and 26 contributed the largest proportion of total somatic SNVs and were similar to previous findings. Both signatures were associated with defective DNA repair [34]. Interestingly, in the hypermutated-cases only we identified Signature 10 (C > A SNVs at TpCpT context), previously shown to correlate with altered activity of DNA polymerase epsilon [34] (termed “ultra-hypermutators” by COSMIC). Indeed, we determined that the two cases with the highest mutation burdens were MMR-intact with mutations in their *POLE* gene: V411L in the exonuclease (proofreading) domain in one case and P286R in the polymerase domain in the other demonstrating the capacity of CGS in identifying clinically useful mutational signatures.

### Genomic evaluation of key driver genes

Recent updates in clinical guidelines, in both Japan and in the US, have made the genomic evaluation of *KRAS*, *NRAS*, and *BRAF* essential for treatment planning. Most mutations in these genes cluster in “hot-spots” (i.e. *KRAS* codon 12, 13; *NRAS* codon 61; *BRAF* codon 600); however, data from large full-gene sequencing projects have identified additional mutations outside these hot-spots (e.g. *KRAS* codon 22, 33, 59, etc.). We compared the distribution of somatic mutation across these key genes between Japanese and US cohorts and with the TCGA (Fig. 2f–h, Additional file 1: Figure S1). While the *KRAS* mutation patterns in different cohorts appeared similar, *BRAF* mutation patterns presented key differences. *BRAF* mutations present in TCGA-CRC samples were predominantly represented by V600E which is often restricted to hypermutated tumors and agrees with previous reports [35–37]. The TCGA database shows that BRAF mutations in non-hypermutated tumors were also significantly more frequent in right-sided tumors. In contrast to previous studies, both Japanese and US-CRC cases had a wide range of non-V600E mutations inside and outside the kinase domain including D594G, a kinase-dead BRAF that can drive tumor progression through interactions with CRAF [38]. In addition, BRAF mutations were found in both left-sided and right-sided tumors (Additional file 1: Table S2). This finding may suggest unique therapeutic strategies for not only right-sided, but also left-sided tumors that were enriched for alternate *BRAF* mutations. Consistent with previous findings in TCGA-CRC cases [39], we found *APC* and *RNF43* truncating mutations mutually exclusive in J-CRC and in US-CRC (Fig. 1) with significant enrichment of *RNF43* alterations, particularly G659 mutations, in MMR-deficient tumors (Additional file 1: Figure S2). Analysis of additional key driver genes showed similar patterns of mutation between Japanese, US, and TCGA cohorts (Additional file 1: Figure S1). Similar to TCGA results, no gene fusions were found in well-characterized driver genes *ALK*, *RET*, or *ROS1*.

### Genomic alterations and tumor aggressiveness

Unlike earlier genomic profiling studies, this study also included clinical outcomes data that was used to determine the relationship between mutation profile and patient outcomes. CRC is a clinically diverse disease and it has been long considered that genomic heterogeneity is vital to understanding this diversity. Tumors can be classified by degree of lymphatic invasion, vascular invasion, histopathological grade, TNM classifications, and tumor stage [21]. We therefore examined the association between gene alterations and clinical features. Among the 415 genes, we found that genes significantly enriched in at least one certain category (*p* < 0.05) were distinctly classified into more aggressive or less aggressive groups (Additional file 1: Figure S3 and Table S3). For example, mutations in genes such as *PTEN*, *SMAD2*, *TGFB2*, and *SRC* implicated in epithelial-mesenchymal transition, metastasis, and cancer progression [40, 41], were enriched in more aggressive groups while the other genes clustered in the less aggressive groups.

### Cluster analysis for Japanese CRC mutations

Several approaches to identify genomic subtypes have been proposed to correlate genomic landscape with clinical features in CRC. Despite differing methods of classification, the hypermutated subtype has commonly emerged across various genomic profiling efforts. In agreement with these findings, we identified a subgroup of 17 Japanese patients with hypermutated tumors as characterized by CGS (Fig. 1). We therefore performed hierarchical clustering of mutations in a subset of genes frequently altered in CRC (*n* = 61 genes) in the Japanese cohort of non-hypermutated patients (*n* = 184 tumors) to further assess the association between gene alterations and clinical features in CRC (Additional file 1: Figure S4). We identified that all patients can be classified into 12 typical clusters (Additional file 1: Figure S4). We further examined associations between each of these clusters with clinicopathological features, such as sex, tumor location, and pathologic stage (Additional file 1: Figure S4B). Of note, patients in Cluster 7 (*n* = 49 tumors) with primary mutated genes *APC* and *TP53* significantly associated with the location of left side (*p* < 0.01), less lymph node metastasis (*p* < 0.05), and less distant metastasis (*p* < 0.05) compared with patients in all other clusters (Additional file 1: Figure S4B). These findings suggest that there are clear associations between mutation spectrum and clinical characteristics of Japanese CRC patients.

**Fig. 3:**
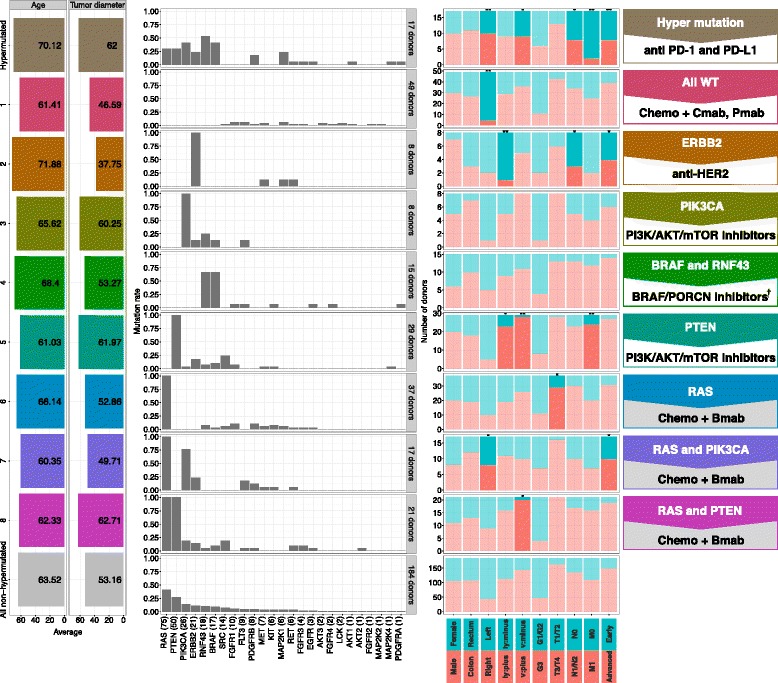
Cluster of 26-gene co-mutation patterns. Cluster analysis was performed on non-hypermutated Japanese CRC samples (*n* = 184 tumors) by using Euclidean distance and Ward’s clustering method and co-mutation patterns of the 26-gene subset with statistical analysis are shown. Mutation rate in each group is shown as a *bar graph* in the *middle panel*. Group-based mean values for age and tumor diameter are shown (*left*) with cluster colors and fraction for clinical information (*right*). *Dark bars* indicate significant difference (*p* < 0.05, two-tailed Fisher’s exact test) to the distribution of all other non-hypermutated donors, *light bars* are non-significant (*p < 0.05, **p < 0.01). *Chemo* chemotherapy; *Cmab* Cetuximab; *Pmab* Panitumumab; *Bmab* Bevacizumab. ^†^Combination therapy with other inhibitors (e.g. anti-EGFR, MEK inhibitors) will be recommended

Additional cluster analysis on a subset of 26 genes associated with targeted therapies either already approved or in late-phase development in Japan (Phase II or III) (Fig. 3, Additional file 1: Figure S5A) identified seven clusters with mutated genes and a single cluster with no mutated genes. Patients with *KRAS* mutations (Clusters 6–8; *n* = 75 tumors) were classified into three clusters, while patients without *KRAS* mutations were classified into either Cluster 1 (*n* = 49 tumors) with “all wild-type” genes or Clusters 2–5 (*n* = 60 tumors) with mutations in actionable driver genes including *ERBB2*, *PIK3CA*, *RNF43*, *BRAF*, and *PTEN*. Patients in Cluster 1 were associated with tumors in the left side (*p* < 0.01), while patients in Cluster 7 (*n* = 17 tumors) with *RAS* and *PIK3CA* mutations were associated with tumors on the right side (*p* < 0.05), consistent with previous reports [42]. Interestingly, patients in Cluster 2 (*n* = 8 tumors) with *ERBB2* mutations were associated with smallest tumor size, significantly less lymphatic invasion (*p* < 0.01) and early stage (*p* < 0.05), while patients in Cluster 5 (*n* = 29 tumors) harboring *PTEN* mutations exhibited significantly more lymphatic (*p* < 0.05) and vascular invasion (*p* < 0.01) with more metastasis.

### Outcome of Stage IV CRC patients and clinical potential of cluster analysis based on CGS platform

**Fig. 4:**
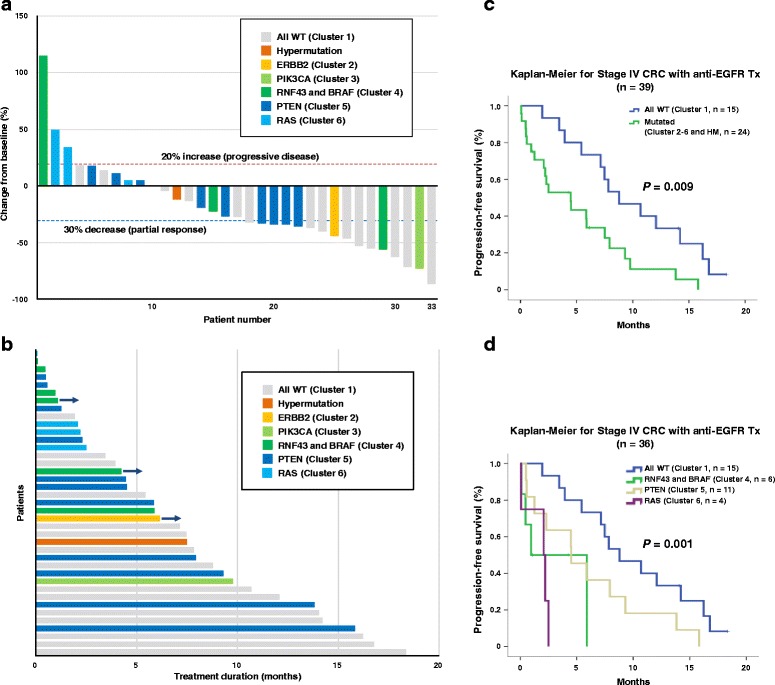
Clinical outcomes of Stage IV patients treated with anti-EGFR therapies. **a** *Waterfall plot* for 33 patients with Stage IV CRC after anti-EGFR targeted therapy in addition to cytotoxic chemotherapy. The *vertical axis* shows the best calculated responses on the basis of measurable lesions in each individual patient. **b** *Swimmers plot* for 39 patients with Stage IV CRC treated with anti-EGFR therapies. The *horizontal axis* shows progression-free survival for each patient. **c**, **d** Kaplan–Meier survival estimates according to genomic subgroups. **c** Progression-free survival was analyzed in 39 patients with Stage IV CRC treated with anti-EGFR therapies. The patients were divided to “All WT (wild type)” (Cluster 1; *n* = 15) or “Mutated” (Clusters 2–8; *n* = 24) based on the cluster analysis with targeted therapy-related 26 genes. **d** Progression-free survival was analyzed for 36 patients with Stage IV CRC treated with anti-EGFR therapies based on subgroups (All WT, cluster 1; RNF and BRAF, cluster 4; PTEN, cluster 5; RAS, cluster 6) by clustering with the 26 genes

Next, we examined clinical outcomes of Stage IV CRC patients to explore the clinical potential of cluster analysis based on CGS for Japanese CRC patients. Kaplan–Meier analysis for patients with Stage IV CRC (*n* = 102, excluded two hypermutated cases) revealed that overall survival rates were significantly different among the subtypes based on cluster analysis on a subset of the 26 genes associated with targeted therapies (Fig. 3, Additional file 1: Figure S5B). The 26 genes included RTK and RAS pathway, such as *KRAS*, *BRAF*, *NRAS*, and *ERBB2*, which have known associations with resistance to anti-EGFR targeted therapies in CRC patients [10]. We therefore hypothesized that the cluster analysis based on the 26 genes estimates the effect of anti-EGFR therapies. Waterfall plot analysis demonstrated the best calculated responses on the basis of measurable lesions in 33 patients treated with anti-EGFR therapies and revealed that all the three patients with progressive disease belong to subgroups with actionable driver mutations (*RNF43* and *BRAF*; Cluster 4 and *RAS*; Cluster 6), but not subgroup of “all wild-type” without actionable mutations (Cluster 1) (Fig. 4a). Moreover, in agreement with previous findings [43], swimmers plot and Kaplan–Meier analysis demonstrated that patients in subgroup of “all wild-type” showed significantly better progression-free survival as compared to patients in subgroups of “mutated” (Clusters 2–6 and hyper-mutated subgroup) (*p* = 0.009) (Fig. 4b and c). Moreover, Kaplan–Meier analysis further demonstrated a significant difference among subgroups when the subgroups with actionable mutations were stratified based on the clustering (*p* = 0.001) (Fig. 4d). These findings indicate clinical potential of clustering based on the 415-gene CGS platform with its ability to estimate the survival of patients with Stage IV CRC treated with targeted therapies.

## Discussion

In the current study, we performed CGS sequencing with a 415-gene panel to probe actionable driver mutations at a very high depth of coverage in the largest series of Japanese patients (*n* = 201 tumors) and evaluated for concordance among independent data obtained from US patients with colon cancer (*n* = 108 tumors) and from the TCGA-CRC WES database (*n* = 224 tumors). We identified overall similarities and some distinct population differences in detecting clinically actionable oncogenic driver events. We correlated mutation burden with DNA mismatch repair status, obtained clear genomic mutational signatures, and identified genomic alteration patterns in Japanese and the US-CRC patients similar to those previously identified by WES by the TCGA. We also found statically significant increases in *ERBB2 APC*, *TP53*, and *NRAS* mutations in Japanese patients as compared with US patients, which may reflect epidemiological differences between the two populations. Interestingly, we found that 11 of 24 *BRAF* mutations occurred outside the hot-spot V600E. Since mutations other than V600E are known to be activating, our results underscore the importance of sequencing all *BRAF* exons to assess the optimal therapeutic approach. Moreover, we report here a novel, significant correlation between *APC* and *TP53* mutations with tumors presented on the left side, emphasizing the utility of CGS sequencing as an invaluable resource for better understanding the genomic landscape of CRC.

To explore the clinical potential of CGS, we performed cluster analysis with the set of clinically actionable genes in CRC (*n* = 26 genes) related to targeted therapies either approved or in late-phase development in Japan and obtained eight typical subgroups in addition to the “hypermutated” subgroup. CRC patients in the “hypermutated” subgroup are expected to benefit most from treatment with immune checkpoint inhibitors. Patients in the “all wild-type” cluster (Cluster 1) may respond best to anti-EGFR therapies, such as Cetuximab and Panitumumab given the lack of contraindicated *KRAS* mutations. However, patients in Clusters 2–5 had driver mutations downstream of the EGFR pathway, suggesting resistance to anti-EGFR therapies and hence better response to therapies targeting *PIK3CA*, *ERBB2*, *RNF43/BRAF*, or *PTEN*. Patients in Clusters 6–8 had *KRAS* mutations and therefore may benefit from chemotherapy + Bevacizumab given their expected resistance to anti-EGFR therapy. Thus, these findings underscore the clinical potential of examining a smaller (26 gene) panel, by which we could identify suitable targeted therapies based on the clustering of actionable gene mutations.

Given the clinical significance of hot-spot *KRAS* mutations (codons 12 and 13) in patients with advanced CRC to anti-EGFR therapy resistance, *KRAS* mutation testing has become mandatory testing in Japanese patients before administering anti-EGFR therapy [44]. Indeed, most of the patients treated with anti-EGFR therapies in this study had been identified not to have hot-spot *KRAS* mutations (codons 12 and 13) and thus considered as *KRAS* wild-type, except for a few patients who had been treated before testing became required. Recent studies have identified alterations in genes downstream of *EGFR* (RTKs and RAS pathway) in addition to hot-spot *KRAS* mutations as likely indicators of primary and secondary resistance to anti-EGFR antibody therapies [10]. We therefore probed the clinical relevance of gene alterations in RTKs and RAS pathway in addition to *KRAS* mutations as identified by CGS in Japanese CRC patients. Interestingly, there were three patients with progressive disease on anti-EGFR therapy and CGS revealed that two out of the three patients had previously unidentified mutations downstream of EGFR emphasizing that hot-spot testing alone is inadequate in guiding therapeutic strategies. Moreover, Kaplan–Meier analysis demonstrated that patients in the subgroup without alterations in RTKs and RAS pathway showed significantly better progression-free survival than patients in subgroups with mutations, although most of the patients had been previously considered as *KRAS* wild-type. Taken together, we have demonstrated that CGS captures broad actionable genomic driver mutations in Japanese patients with advanced CRC satisfying a currently unmet critical need to better guide personalized therapeutic approaches in Japan.

## Conclusions

We demonstrate concordance of CGS between Japanese and US patients with CRC and with WES in the TCGA database. We further illustrate how CGS testing captures broad actionable genomic driver mutations as well as high mutational burden and highlight its potential to impact clinical outcomes of patients. These findings emphasize the clinical potential of CGS for patients with CRC in Japan and warrant further clinical investigation through prospective randomized clinical trials to confirm the application.

## Additional file

Additional file 1: Figure S1. Location of genetic aberrations for Japanese and US patients, and TCGA samples. Mutations in (**A**) APC, (**B**) ERBB2, (**C**) TP53, (**D**) NRAS, and (**E**) KRAS for Japanese patients (*n* = 201), US patients (*n* = 108), and TCGA samples (*n* = 224) were aligned to protein domains. The number of mutations at each given amino acid were plotted in corresponding *pie graphs*. As shown, KRAS G12 were the highest frequency mutations. Patient samples were further plotted by mutation status (**F**) KRAS-hypermutated and (**G**) KRAS-non-hypermutated. **Figure S2.** Correlation of RNF43 mutations with MMR. (**A**) The frequencies of APC and RNF43 mutations were determined by MMR phenotype. Statistical significance was determined by Fisher’s exact test. (**B**) Mutation mapper analysis identified G659 as most frequently altered in MMR-D cases. **Figure S3.** Gene-based statistical analysis for clinical information. Genes were filtered based on Fisher’s exact test (*p* < 0.05). Cell values are log odds ratios colored from *blue* to *red*. Dendrograms were created by Euclidean distance and Ward’s method. Less (*blue*) or more (*red*) aggressive factors of seven clinical variables are shown: lymphatic invasion (ly), vascular invasion (v), histopathological grade (G), TNM classifications (T, N, and M), and tumor stage. **Figure S4.** Cluster of 61-gene co-mutation patterns. (**A**) Cluster analysis was performed on non-hypermutated Japanese CRC samples (*n* = 184 tumors) by using Euclidean distance and Ward’s clustering method (closest distance to common mutated genes are colored *yellow* to *blue*). (**B**) Co-mutated gene patterns of the 61-gene set with statistical analysis. Mutation rate in each group is shown as a *bar graph* in the *middle panel*. Group-based mean values for age and tumor diameter are shown (*left*) with cluster colors and fraction for clinical information (*right*). *Dark bars* indicate significant difference (*p* < 0.05, two-tailed Fisher’s exact test) to the distribution of all other non-hypermutated donors, *light bars* are non-significant (***p* < 0.01, **p* < 0.05). **Figure S5.** Data complementary to Fig. 3. (**A**) Cluster analysis was performed on non-hypermutated Japanese CRC samples (*n* = 184 tumors) by using Euclidean distance and Ward’s clustering method (closest distance to common mutated genes are colored *yellow* to *blue*). (**B**) Kaplan–Meier survival estimates according to genomic subgroups. Overall survival was analyzed in 102 patients with Stage IV CRC treated with anti-EGFR therapies. The patients were divided to “All WT (wild type)” (Cluster 1; *n* = 25) or “Mutated” (Clusters 2–8; *n* = 77) based on the cluster analysis with targeted therapy-related 26 genes. **Table S1.** The 415-gene list for the CGS platform. **Table S2.** BRAF mutation and tumor location (J-CRC, *n* = 201). **Table S3.** Raw data for gene-based statistical analysis for clinical information. **Table S4.** Clinicopathological characteristics of 201 CRC patients. (PDF 1435 kb)

## Abbreviations

CGS: Comprehensive genomic sequencing
CNV: Copy number variation
FFPE: Formalin-fixed, paraffin embedded
MMR-D: Mismatch repair deficiency
SNV: Single nucleotide variant
TCGA: The Cancer Genome Atlas
WES: Whole exome sequencing
