# Supplementary material for: Genomic landscape of colorectal cancer in Japan: clinical implications of comprehensive genomic sequencing for precision medicine
Source: Genome Med. 2016 Dec 22;8:136. doi: 10.1186/s13073-016-0387-8 (PMC5180401; doi:10.1186/s13073-016-0387-8)
Supplement: Additional file 1: Figure S1. — Location of genetic aberrations for Japanese and US patients, and TCGA samples. Mutations in (A) APC, (B) ERBB2, (C) TP53, (D) NRAS, and (E) KRAS for Japanese patients (n = 201), US patients (n = 108), and TCGA samples (n = 224) were aligned to protein domains. The number of mutations at each given amino acid were plotted in corresponding pie graphs. As shown, KRAS G12 were the highest frequency mutations. Patient samples were further plotted by mutation status (F) KRAS-hypermutated and (G) KRAS-non-hypermutated. Figure S2. Correlation of RNF43 mutations with MMR. (A) The frequencies of APC and RNF43 mutations were determined by MMR phenotype. Statistical significance was determined by Fisher’s exact test. (B) Mutation mapper analysis identified G659 as most frequently altered in MMR-D cases. Figure S3. Gene-based statistical analysis for clinical information. Genes were filtered based on Fisher’s exact test (p < 0.05). Cell values are log odds ratios colored from blue to red. Dendrograms were created by Euclidean distance and Ward’s method. Less (blue) or more (red) aggressive factors of seven clinical variables are shown: lymphatic invasion (ly), vascular invasion (v), histopathological grade (G), TNM classifications (T, N, and M), and tumor stage. Figure S4. Cluster of 61-gene co-mutation patterns. (A) Cluster analysis was performed on non-hypermutated Japanese CRC samples (n = 184 tumors) by using Euclidean distance and Ward’s clustering method (closest distance to common mutated genes are colored yellow to blue). (B) Co-mutated gene patterns of the 61-gene set with statistical analysis. Mutation rate in each group is shown as a bar graph in the middle panel. Group-based mean values for age and tumor diameter are shown (left) with cluster colors and fraction for clinical information (right). Dark bars indicate significant difference (p < 0.05, two-tailed Fisher’s exact test) to the distribution of all other non-hypermutated donors, light bars are non-significant [file 13073_2016_387_MOESM1_ESM.pdf]

Figure S1

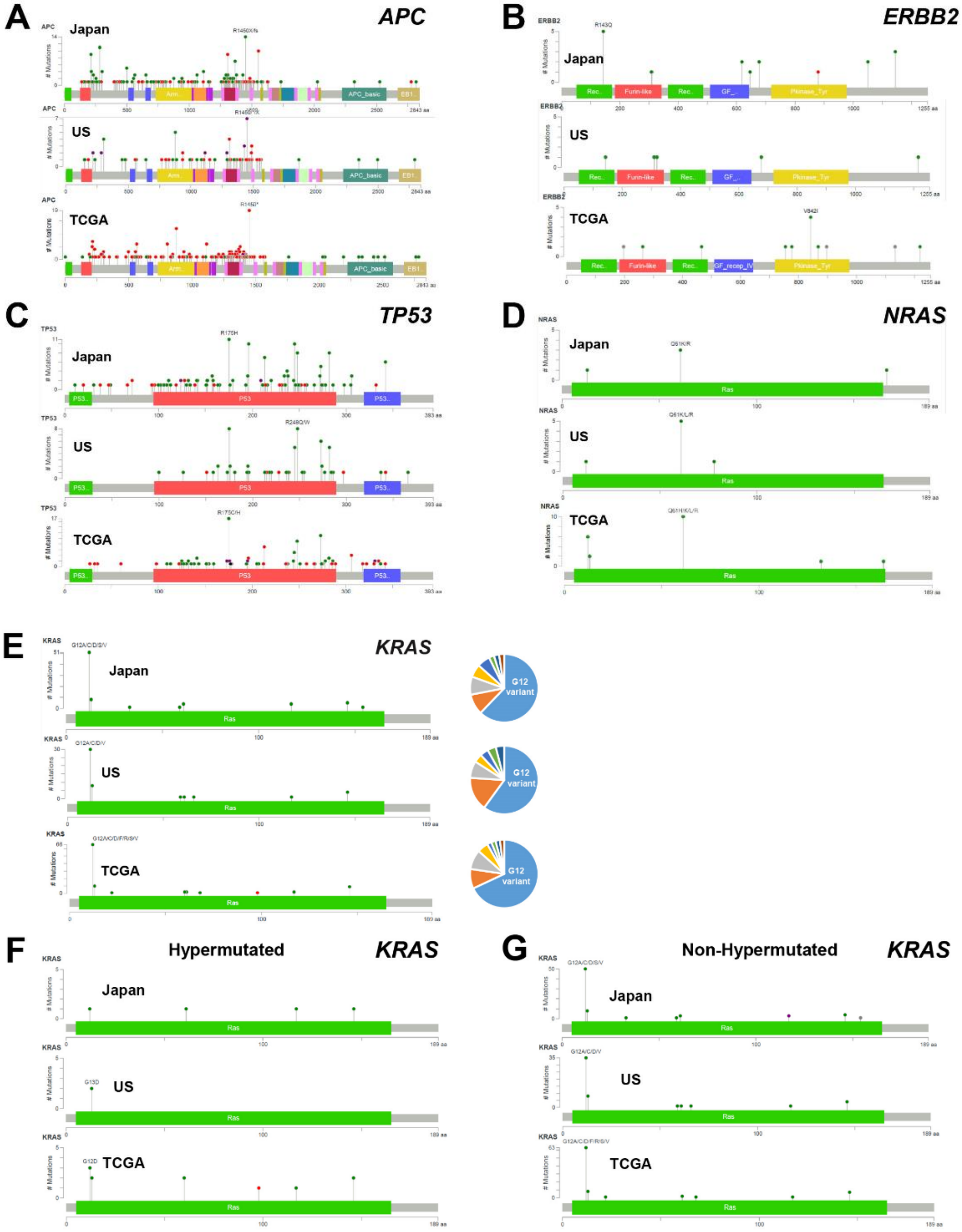

Figure S1. Location of genetic aberrations for Japanese and US patients, and TCGA samples. Mutations in (**A**) APC (**B**) ERBB2 (**C**) TP53 (**D**) NRAS (**E**) KRAS for Japanese patients (n = 201), US patients (n = 108) and TCGA samples (n = 224) were aligned to protein domains. The number of mutations at each given amino acid were plotted in corresponding pie

graphs. As shown, KRAS G12 were the highest frequency mutations. Patient samples were further plotted by mutation status (**F**) KRAS-hypermutated, (**G**) KRAS-non-hypermutated.

Figure S2

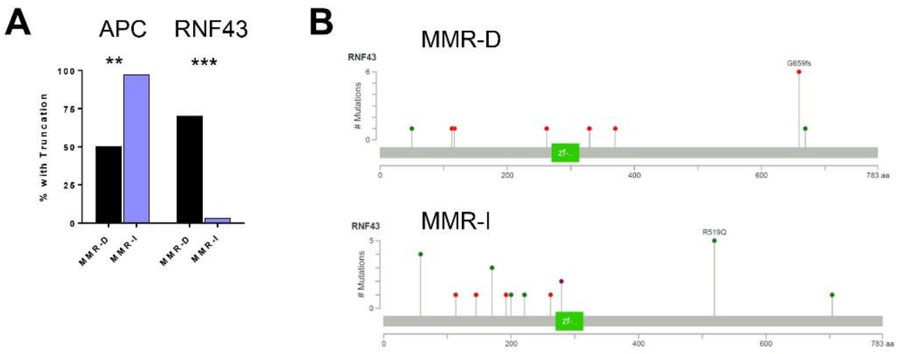

Figure S2. Correlation of RNF43 mutations with MMR. (**A**) The frequencies of APC and RNF43 mutations were determined by MMR phenotype. Statistical significance was determined by Fisher's exact test. (**B**) Mutation mapper analysis identified G659 as most frequently altered in MMR-D cases.

Figure S3

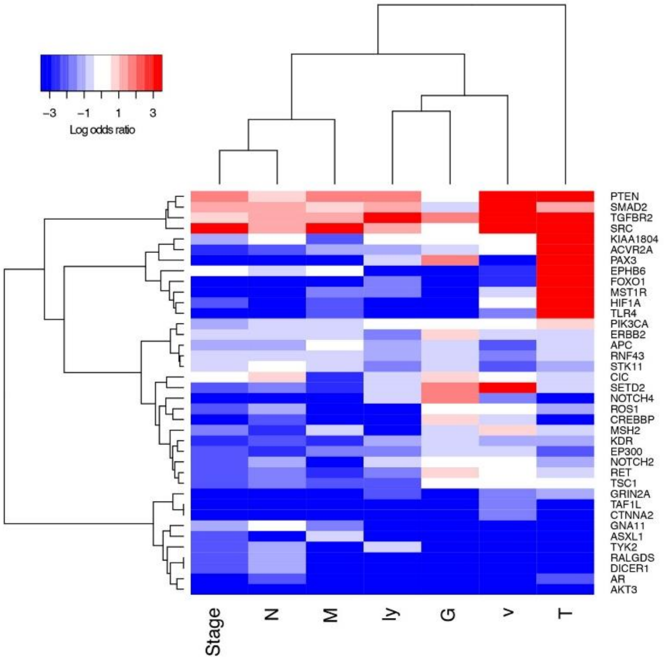

Figure S3. Gene-based statistical analysis for clinical information. Genes were filtered based on Fisher exact test ($P < 0.05$). Cell values are log odds ratios colored from blue to red. Dendrograms were created by Euclidean distance and Ward's method. Less (blue) or more (red) aggressive factors of seven clinical variables are shown: lymphatic invasion (ly), vascular invasion (v), histopathological grade (G), TNM classifications (T, N, and M), and tumor stage.

Figure S4

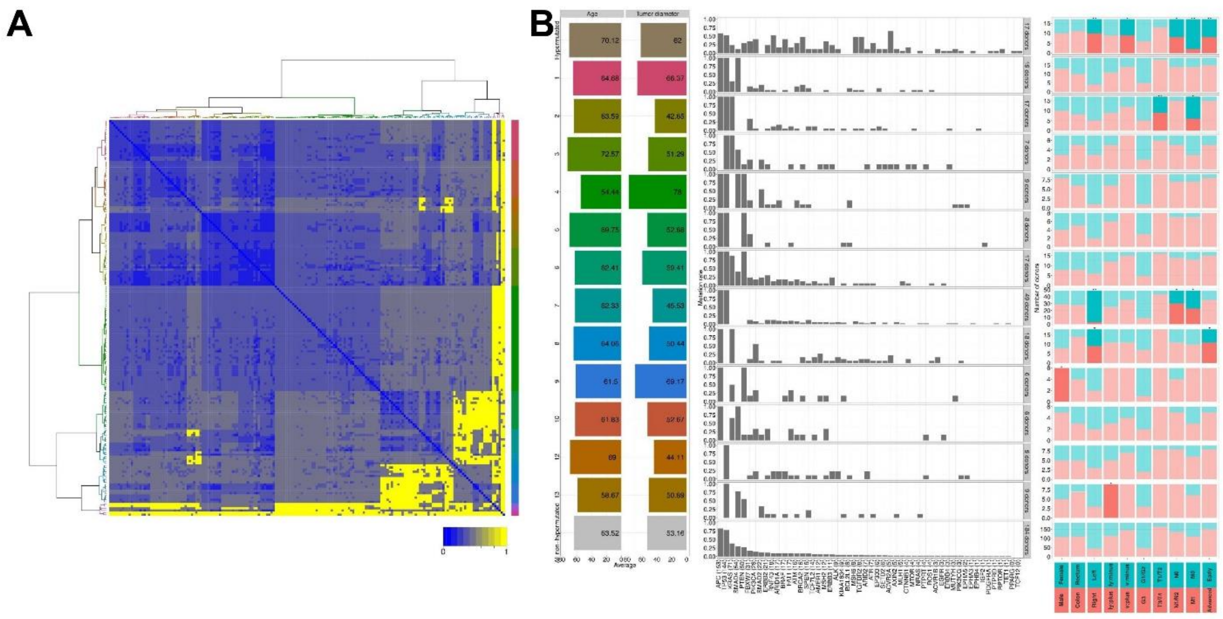

Figure S4. Cluster of 61-gene co-mutation patterns. (**A**) Cluster analysis was performed on non-hypermutated Japanese CRC samples (n = 184 tumors) by using Euclidean distance and Ward's clustering method (closest distance to common mutated-genes are colored yellow to blue). (**B**) Co-mutated gene patterns of 61-gene set with statistical analysis. Mutation rate in each group is shown as a bar graph in the middle panel. Group-based mean values for age and tumor diameter are shown (left) with cluster colors and fraction for clinical information (right). Dark bars indicate significant difference ($P < 0.05$, two-tailed Fisher's exact test) to the distribution of all other non-hypermutated donors, light bars are non-significant (**$P < 0.01$, *$P < 0.05$).

Figure S5

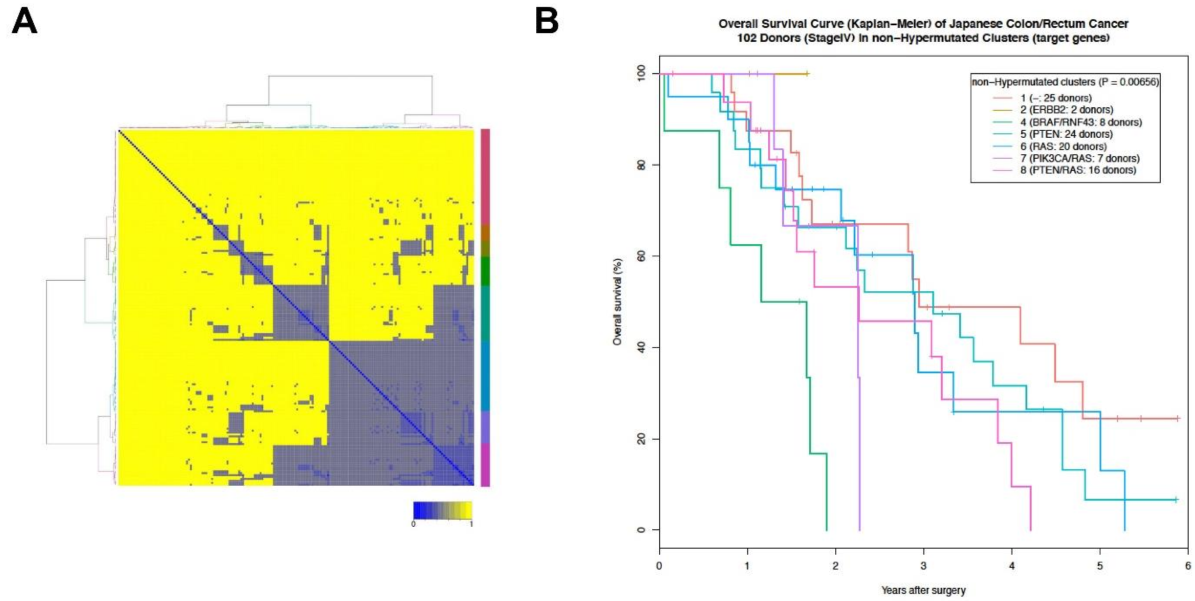

Figure S5. Data Complementary to Figure 3. (A) Cluster analysis was performed on non-hypermutated Japanese CRC samples (n = 184 tumors) by using Euclidean distance and Ward's clustering method (closest distance to common mutated-genes are colored yellow to blue). (B) Kaplan-Meier survival estimates according to genomic subgroups. Overall survival was analyzed in 102 patients with Stage IV CRC treated with anti-EGFR therapies. The patients were divided to "All WT (wild type)" (cluster 1; n = 25) or "Mutated" (cluster 2-8; n = 77) based on the cluster analysis with targeted therapy-related 26 genes.

Table S1

Table S1. 415 gene list for the comprehensive genomic sequencing platform

GENE LIST							
ABL1	CASP8	EP300 (P300)	FOXL2	KAT6B (MYST4)	MYCL1	PIM1	SOX11
ABL2 (ARG)	CBFB	EPHA3	FOXO1	KDM5A (JARID1A)	MYCN	PKHD1	SOX2
ACVR1B	CBL	EPHA5	FOXP1	KDM5C	MYD88	PLAG1	SOX9
ACVR2A	CCND1 (Cyclin D1)	EPHA7	G6PD	KDM6A	MYH11	PLCG1	SPEN
AFF1	CCND2 (Cyclin D2)	EPHB1	GATA1	KDR (VEGFR2)	MYH9	PML	SPOP
AKT1	CCND3 (Cyclin D3)	EPHB4	GATA2	KEAP1	NBN	PMS1	SRC
AKT2	CCNE1 (Cyclin E1)	EPHB6	GATA3	KIAA1804 (MLK4)	NCOA1	POT1	STAG2
AKT3	CD70 (TNFSF7)	ERBB2 (HER2)	GDNF	KIT	NCOA2	PPARG	STAT3
ALK	CD79A	ERBB3 (HER3)	GID4	KLF5	NCOA4	PPP2R1A	STAT4
AMER1 (FAM123B)	CD79B	ERBB4 (HER4)	GNA11	KLF6	NF1	PPP6C	STK11(LKB1)
APC	CDC73 (HRPT1)	ERCC1 (RAD10)	GNA13	KLHL6	NF2 (Merlin)	PRDM1 (BLIMP1)	STK36(FU)
AR	CDH1 (E-Cadherin)	ERCC2 (XPD)	GNAQ	MLL (KMT2A)	NFE2L2 (NRF2)	PRKAR1A	SUFU
ARAF	CDH11 (Cadherin 11)	ERCC3 (XPB)	GNAS	MLL2 (KMT2D)	NFKB1 (NFKB-p105)	PRKDC	SYK
ARFRP1 (ARP1)	CDH2 (N-Cadherin)	ERCC4 (XPF)	GPC6	KRAS	NFKB1A	PSIP1	TAF1
ARID1A	CDH5 (VE-Cadherin)	ERCC5	GPR124	LAMP1	NFKB2 (NFKB)	PTCH1	TAF1L
ARID2	CDK12	ERG	GRIK3	LCK	NIN	PTEN	TAL1(SCL)
ARNT (HIF-1β)	CDK4	ESR1	GRIN2A	LPP	NKX2-1 (TTF1)	PTGS2 (COX2)	TBX22
ASCL4	CDK6	ETS1	GSK3B	LRP1B	NLRP1	PTPRD	TCF12
ASXL1	CDK8	ETV1	HCAR1	LTF	Notch1	PTPRT	TCF3
ATM	CDKN1A	ETV4	HCN1	MAGEA1	NOTCH2	RAC1	TCF7L1
ATR	CDKN1B (p27)	EXT1	HGF	MAGI1	NOTCH4	RAF1 (CRAF)	TCF7L2
ATRX	CDKN2A (INK4)	EXT2	HIF1A	MALT1	NPM1	RALGDS	TCL1A
AURKA	CDKN2B (INK4B)	EZH2	HLF	MAML2	NRAS	RARA	TET1
AURKB	CDKN2C (INK4C)	FAM46C	HNF1A	MAP2K1 (MEK1)	NTRK1	RASA1	TET2
AURKC	CEBPA	FANCA	HNF4A	MAP2K2 (MEK2)	NTRK2	RB1	TET3
AXIN2	CHEK1	FANCC	HRAS	MAP2K4 (MEK4)	NTRK3	REL	TGFBR2
AXL (UFO)	CIC	FANCE	HSP90AA1	MAP3K1 (MEKK1)	NUMA1	RET	TGM7
BAP1	CREBBP	FANCF	HSP90AB1	MAP3K7 (TGF1A)	NUP214	RHOH	TLR4
BARD1	CRKL	FANCG	ICK	MAPK1 (ERK2)	NUP93	RICTOR	TNFAIP3(A20)
BCL10	CRLF2	FANCL	IDH1	MAPK8 (JNK1)	NUP98	RNASEL	TNFRSF14
BCL11A (ZNF856)	CRTC1	FAS (APO-1)	IDH2	MBD1	PAK3	RNF2	TNK2(ACL1)
BCL11B	CSF1R	FAT1	IGF1R	MCL1	PALB2	RNF43	TOP1
BCL2	CSMD3	FBXW7 (BAF250)	IGF2	MDM2	PARP1	ROS1	TP53(P53)
BCL2L1 (BCL-X)	CTCF	FGF10	IGF2R	MDM4	PAX3	RPS6KA2	TP63
BCL2L2 (BCL-W)	CTNNA1 (α1-Catenin)	FGF14	IKBKB (IKKKB)	MED12	PAX5	RPTOR (RAPTOR)	TPR
BCL6	CTNNA2 (α2-Catenin)	FGF19	IKBKE	MEF2B	PAX7	RRM1	TRIM33(TIF1γ)
BCL9	CTNNB1 (β-Catenin)	FGF23	IKZF1 (IKAROS)	MEN1	PAX8	RUNX1 (AML1)	TRIP11
BCOR	CUX1	FGF3	IL2	MET	PBRM1	RUNX1T1 (ETO)	TRRAP
BCORL1	CYLD	FGF4	IL21R	MITF	PBX1	SAMD9	TSC1
BIRC2 (IAP2)	DAXX	FGF6	IL6ST (CD130)	MLH1	PDGFRA	SDHB	TSC2
BIRC3 (AIP1)	DCC	FGFR1	IL7R	MMP2	PDGFRB	SETD2	TSHR
BLM	DDB2	FGFR2	INHBA	MN1	PDK1	SF3B1	TYK2
BLNK	DDIT3 (GADD153)	FGFR3	IRF4	MPL	PGAP3	SGK1	UBR5
BMPR1A	DDR1	FGFR4	IRS2	MRE11A	PHOX2B	SH2D1A	USP9X
BRAF	DDR2	FH	ITGA10	MSH2	PIK3C2B (PI3K-C2β)	SLIT2	VHL
BRCA1	DICER1	FLCN	ITGB2	MSH6	PIK3CA (p110α)	SMAD2	WISP3
BRCA2	DNMT3A	FLI1	ITGB3	MST1R (RON)	PIK3CB (PIK3C1)	SMAD4	WT1
BRIP1 (FANCJ)	DOT1L	FLT1 (VEGFR1)	JAK1	MTOR	PIK3CD (p110δ)	SMARCA4	XPC
BTK	DST	FLT3 (STK1)	JAK2	MTR	PIK3CG (p110γ)	SMARCB1	XPO1(CRM1)
BUB1B	EDNRB	FLT4 (VEGFR3)	JAK3	MTRR	PIK3R1 (GRB1)	SMO	ZNF384
C11orf30 (EMSY)	EGFR	FN1	JUN	MUTYH	PIK3R2 (p85)	SOCS1	ZNF521(EVI3)
CARD11	EML4	FOXA1 (HNF3A)	KAT6A (MOZ)	MYC	PIK3R3 (p55γ)	SOX10	

Table S2

Table S2. BRAF mutation and tumor location (J-CRC, n = 201)

		BRAF mutation			*P* value
		Absent	Present		
			V600E	Non-V600E	
Location	Rt : Lt	40 : 137	12 : 1	3 : 8	< 0.001

Table S3

Table S3. Raw data for gene-based statistical analysis for clinical information

Gene/Categoı	ly_logOR	ly_P	v_logOR	v_P	G_logOR	G_P	T_logOR	T_P	N_logOR	N_P	M_logOR	M_P	Stage_logOR	Stage_P
ACVR2A	-1.284605972	0.3755518	0.155294596	1	-0.507606552	1	4	1	-2.102652993	0.1184945	-1.183457306	0.392881	-2.8172955	0.0445392
AKT3	-4	0.1476004	-4	0.04632929	-4	1	-3.067989641	0.206106	-4	0.06985032	-4	0.16043	-4	0.0333215
APC	-1.295281341	0.06725693	-2.251591667	0.02947482	-0.524142289	0.3794865	-0.94670123	0.5352907	-1.064633493	0.183836	-0.108808085	1	-1.24029055	0.2098898
AR	-4	0.05595839	-4	0.009673149	-4	0.5685854	-2.073359256	0.2933471	-2.494977387	0.1734744	-4	0.06346683	-3.192881205	0.0882469
ASXL1	-4	0.1476004	-4	0.04632929	-4	1	-3.067989641	0.206106	-4	0.06985032	-0.575097255	1	-2.158068734	0.3362438
CIC	-0.689931225	0.6776591	0.485955375	1	0.517781619	0.6519424	-0.738161603	0.5036991	0.880398721	1	-2.967014481	0.03942998	0.185437795	1
CREBBP	-4	0.05595839	-0.858887698	0.5228592	0.508501673	1	-4.13054535	0.03162376	-2.494977387	0.1734744	-4	0.06346683	-3.192881205	0.0882469
CTNNA2	-4	0.1476004	-1.861391009	0.3884533	-4	1	-3.067989641	0.206106	-4	0.06985032	-4	0.16043	-4	0.0333215
DICER1	-4	0.1476004	-4	0.04632929	-4	1	-3.067989641	0.206106	-1.471616719	0.4627584	-4	0.16043	-2.158068734	0.3362438
EP300	-1.718624356	0.2072926	-0.875775107	0.6123848	-0.839164466	1	-2.132899965	0.1291324	-2.546382759	0.04407598	-1.616609672	0.2215466	-2.228140107	0.0777698
EPHB6	-4	0.007825243	-2.506705186	0.06944359	-4	0.3286899	4	1	-0.899239748	0.6102924	0.013347912	1	-0.144645866	1
ERBB2	-1.547734362	0.03030159	-0.598267096	0.4085125	0.569845889	0.4343159	-0.514269208	0.7068149	-0.523499272	0.4429833	-0.799511968	0.2449152	-0.941590953	0.2322211
FOXO1	-1.689344383	0.5601184	-2.890523361	0.1196416	-4	0.5685854	4	1	-4	0.01803827	-4	0.06346683	-4	0.0058587
GNA11	-4	0.05595839	-4	0.009673149	-4	0.5685854	-4.13054535	0.03162376	-0.46758329	1	-1.589458734	0.565658	-1.157180105	0.4602422
GRIN2A	-2.29240312	0.3000779	-1.888440865	0.2065227	-4	0.5741469	-1.486263082	0.3714303	-4	0.004584312	-4	0.02489583	-4	0.0010034
HIF1A	-4	0.02102304	-0.267667232	1	-4	0.5741469	4	1	-3.106993866	0.05840014	-2.19169173	0.3040929	-2.192437345	0.1560693
KDR	-1.284605972	0.3755518	-1.297958493	0.2979088	-0.507606552	1	-1.066379323	0.4412713	-2.102652993	0.1184945	-2.625350198	0.1593122	-2.8172955	0.0445392
KIAA1804	-0.36480729	0.7355785	-0.042518163	1	-0.318018823	1	4	0.6003606	-0.484832559	0.7019028	-2.482898902	0.03149532	-1.207531084	0.3705754
MSH2	-3.168830988	0.001479283	0.501234287	1	-0.864102434	0.7343846	-0.769894646	0.623339	-2.657669185	0.003152127	-0.6095117	0.5484971	-1.80321219	0.0480152
MST1R	-1.689344383	0.5601184	-0.858887698	0.5228592	-4	0.5685854	4	1	-4	0.01803827	-1.589458734	0.565658	-3.192881205	0.0882469
NOTCH2	-0.681973376	0.6403178	-0.267667232	1	-0.083811082	1	-1.486263082	0.3714303	-1.4910469	0.2885487	-4	0.02489583	-2.192437345	0.1560693
NOTCH4	-0.674200064	1	-1.861391009	0.3884533	1.512363437	0.4547398	-3.067989641	0.206106	-4	0.06985032	-4	0.16043	-4	0.0333215
PAX3	-0.674200064	1	-4	0.04632929	1.512363437	0.4547398	4	1	-4	0.06985032	-4	0.16043	-4	0.0333215
PIK3CA	0.00884827	1	-0.478026836	0.4543292	0.388588531	0.6304497	0.622586499	0.7438598	-0.925794643	0.1548468	-0.665383445	0.287826	-1.483883162	0.030135
PTEN	1.515538374	0.006140614	3.235593352	0.000202584	-0.216974899	0.8505134	3.007111521	0.01621924	0.71186049	0.2623133	1.861017605	0.000662422	1.722303384	0.0315458
RALGDS	-4	0.1476004	-4	0.04632929	-4	1	-3.067989641	0.206106	-1.471616719	0.4627584	-4	0.16043	-2.158068734	0.3362438
RET	-1.718624356	0.2072926	0.485955375	1	0.517781619	0.6519424	-0.738161603	0.5036991	-1.51104536	0.192913	-2.967014481	0.03942998	-2.228140107	0.0777698
RNF43	-1.099150113	0.1327786	-1.732161732	0.02971689	-0.890653884	0.4107714	-0.804541778	0.4210965	-0.513259682	0.5751986	-0.988777182	0.2068802	-0.856077407	0.334904
ROS1	-4	0.02102304	-0.267667232	1	-0.083811082	1	-1.486263082	0.3714303	-1.4910469	0.2885487	-4	0.02489583	-2.192437345	0.1560693
SETD2	-0.689931225	0.6776591	4	0.3424384	1.553256291	0.1838005	-0.738161603	0.5036991	-1.51104536	0.192913	-2.967014481	0.03942998	-2.228140107	0.0777698
SMAD2	1.218889637	0.1600386	4	0.00475656	-0.743048298	0.4471453	1.474560124	0.4756843	1.322558703	0.1993075	0.592643125	0.4897586	1.293901317	0.378267
SRC	1.283285898	0.2541192	4	0.04243197	0.194803177	0.7608989	4	0.3702242	1.191646026	0.3597853	4	0.000929095	4	0.0762531
STK11	-1.752870168	0.0142476	-2.143697248	0.002892355	-0.549758652	0.6003726	-1.201057302	0.2421052	-0.268643472	0.7895144	-0.640211006	0.3472434	-0.630047367	0.54
TAF1L	-4	0.1476004	-1.861391009	0.3884533	-4	1	-3.067989641	0.206106	-4	0.06985032	-4	0.16043	-4	0.0333215
TGFBR2	4	0.02408589	4	0.2039284	1.574646504	0.2088114	4	0.6017747	1.386330888	0.6836729	1.049229963	0.4780347	0.688603815	1
TLR4	-4	0.02102304	-1.888440865	0.2065227	-4	0.5741469	4	1	-3.106993866	0.05840014	-2.19169173	0.3040929	-3.818710048	0.0204246
TSC1	-2.344100875	0.05653853	-0.274080715	0.685341	-0.085694037	1	-0.237944303	1	-1.531562654	0.2120499	-2.240266692	0.06206673	-2.265268966	0.0397059
TYK2	-0.674200064	1	-4	0.04632929	-4	1	-3.067989641	0.206106	-1.471616719	0.4627584	-4	0.16043	-2.158068734	0.3362438

P-value < 0.05

Table S4

Table S4. Clinicopathological characteristics of colorectal cancer 201 patients

Variable	
Age (years)[a]	65 (30–94)
Sex	
Male : Female	84 : 117
Tumor size (mm)[a]	50 (7–155)
Location	
Colon : Rectum	120 : 81
T category	
T1: T2 : T3 : T4a : T4b	6 : 18 : 97 : 64 : 16
Histopathological grading	
G1 : G2 : G3	0.553402778
Lymphatic invasion	
Absence : Presence	79 : 122
Venous invasion	
Absence : Presence	48 : 153
N category	
N0 : N1a : N1b : 2a : 2b	58 : 34 : 29 : 41 : 39
M category	
M0 : M1a : M1b	88 : 63 : 50
Stage	
I : IIA : IIB : IIC : IIIA : IIIB : IIIC : IVA : IVB	11 : 26 : 5 : 1 : 11 : 23 : 11 : 63 : 50

[a] Data are expressed as median (range)
